# The Serum Glycome to Discriminate between Early-Stage Epithelial Ovarian Cancer and Benign Ovarian Diseases

**DOI:** 10.1155/2014/238197

**Published:** 2014-08-12

**Authors:** Karina Biskup, Elena Iona Braicu, Jalid Sehouli, Rudolf Tauber, Véronique Blanchard

**Affiliations:** ^1^Institute of Laboratory Medicine, Clinical Chemistry and Pathobiochemistry, Charité Medical University, Augustenburger Platz 1, 13353 Berlin, Germany; ^2^Department of Gynecology, Charité Medical University, Augustenburger Platz 1, 13353 Berlin, Germany

## Abstract

Epithelial ovarian cancer (EOC) is the sixth most common cause of cancer deaths in women because the diagnosis occurs mostly when the disease is in its late-stage. Current diagnostic methods of EOC show only a moderate sensitivity, especially at an early-stage of the disease; hence, novel biomarkers are needed to improve the diagnosis. We recently reported that serum glycome modifications observed in late-stage EOC patients by MALDI-TOF-MS could be combined as a glycan score named GLYCOV that was calculated from the relative areas of the 11 N-glycan structures that were significantly modulated. Here, we evaluated the ability of GLYCOV to recognize early-stage EOC in a cohort of 73 individuals comprised of 20 early-stage primary serous EOC, 20 benign ovarian diseases (BOD), and 33 age-matched healthy controls. GLYCOV was able to recognize stage I EOC whereas CA125 values were statistically significant only for stage II EOC patients. In addition, GLYCOV was more sensitive and specific compared to CA125 in distinguishing early-stage EOC from BOD patients, which is of high relevance to clinicians as it is difficult for them to diagnose malignancy prior to operation.

## 1. Introduction

Ovarian cancer, a frequent form of gynecological malignancy, is the sixth most common cause of cancer deaths in women. Serous tumor of epithelial origin, accounting for about 80% of all tumor types, is the most frequent form of ovarian cancers [1, 2]. Based on its prevalence and mortality rate, epithelial ovarian cancer (EOC) is the ovarian cancer with the poorest prognosis of all primary tumor subtypes [2]. The mortality of ovarian cancer patients is as high as 56% per year because diagnosis occurs mostly at an advanced stage [3]. This is because the majority of patients already have metastases at the time of diagnosis, which has remained unchanged in the past 30 years [4]. For patients diagnosed in stage I, the 5-year survival rate ranges from 50 to 90% whereas this rate drops to only 25% when the diagnosis occurs at a later stage [5–7]. The poor detection of ovarian cancer in early stages is attributed to the lack of symptoms and the absence of serum markers that are able to detect the disease at its onset. The search for tumor markers enabling the early detection of EOC is of high importance to improve the clinical outcome.

Despite decades of research to find suitable serum-based diagnostic markers for ovarian cancer, CA125 remains the only reliable marker currently used in the routine diagnostics. This marker is useful for disease monitoring, especially in postoperative women. It helps the clinician to assess the response to chemotherapy and to monitor residual tumor recurrence [8–11]. However, CA125 displays low sensitivity for early-stage patients (stages I/II), which is estimated to be about 65% when the specificity is set to 97% [12, 13]. CA125 is not recommended to screen asymptomatic women as it is overexpressed in normal tissues under several physiological and pathological conditions such as pregnancy, endometriosis, liver cirrhosis, or colon cancer [14–16]. Recently it has been reported that HE4, a new biomarker for ovarian cancer, has a better diagnostic efficiency than CA125 only in premenopausal women and women with a pelvic mass but is not suitable to check asymptomatic population [17–20]. In addition, preoperative blood HE4 and CA125 help to predict the surgical outcome of primary EOC patients as elevated values correlate with poor surgical outcome in terms of macroscopically residual tumor mass and resistance to chemotherapy [21].

Glycosylation is a protein post-translational modification that plays a major role in many biological and pathological processes [22–24]. Moreover, glycome modulations are observed in the course of diseases in tissues, extracellular fluids and serum. Therefore, glycan alterations in blood have been proposed as biomarkers for diagnostic, monitoring, and prognostic purposes in several diseases. Increased agalactosylation and sialylation of immunoglobulins have been correlated with the development of rheumatoid arthritis [25]. On the contrary, the improvement of the disease during pregnancy was associated with increased IgG galactosylation [26]. Increase in serum fucosylation, antennarity, and sialylation, especially of monofucosylated triantennary trisialylated N-glycan, is observed in severe inflammations as well as in cancer [27–31]. The modulations of N-glycans appear to be cancer-specific: increase of disialylated monofucosylated tetraantennary N-glycans is commonly observed in breast cancer patients [27]; decrease of asialylated and monosialylated monofucosylated monogalactosylated biantennary was described for esophageal adenocarcinoma [30]; increase of agalactosylated biantennary glycans is accompanied by a decrease of high-mannose and monoantennary complex-type N-glycans for gastric cancer [31]. In a recent study, we investigated the serum N-glycan profile of a cohort composed of 63 preoperative EOC patients, mostly in stages III and IV, and age-matched healthy volunteers using MALDI-TOF mass spectrometry. We proposed a novel glycan biomarker called GLYCOV that is able to diagnose late-stage EOC better than CA125, the routine serum marker. In this study, we compared the ability of both markers to differentiate between early-stage EOC and benign ovarian diseases (BOD) or healthy controls.

## 2. Patients and Methods

All chemicals were purchased from Sigma-Aldrich, MO, unless stated otherwise.

### 2.1. Patient Population

Serum samples from preoperative primary ovarian cancer patients were collected between 03/2003 and 01/2012 prior to surgery at the Charité Medical University (Berlin, Germany). The Ethics Committee approved the use of the samples (EA4/073/06) for this study. A total of 20 primary serous early-stage patients (FIGO: stage 1, *n* = 10; stage 2, *n* = 10; grading: grade I, *n* = 1; grade II, *n* = 10; grade III, *n* = 9), 20 patients suffering from benign ovarian diseases, and 33 age-matched healthy controls were enrolled in this study. The diagnosis of EOC was histologically confirmed. Patients' data is summarized in Table 1. The patient's informed consent was obtained prior to surgery or during subsequent treatment, sample collection, and documentation of clinical and surgical data. A validated documentation system was used to record surgical data. The tumor pattern was intraoperatively prospectively assessed based on the surgical procedures performed and through a systematic interview of the surgical team. All histological findings and associated data were postoperatively entered into a validated documentation system, specifically developed for ovarian neoplasms [32–36]. The menopausal status was not provided in this study.

### 2.2. Serum Collection

Blood was collected within the Tumor Bank Ovarian Cancer project (http://www.toc-network.de/) using serum tubes containing clot activators (Vacutainer, BD, Medical-Pharmaceutical System, Franklin Lakes, NJ). Collected blood was clotted for 30 min to 2 h at room temperature and serum was separated by centrifugation at 1200 g for 15 minutes. Serum was aliquoted and stored at −80°C until the time of analysis.

### 2.3. Release, Isolation, and Permethylation of N-Glycans

N-Glycans were released and isolated from serum samples as described in Biskup et al. [28]. Briefly, 10 *μ*L of serum was dissolved in phosphate buffer (pH 6.5), reduced with dithioerythritol, and alkylated with iodoacetamide. After incubation the reaction was stopped with addition of an excess of dithioerythritol. N-Glycans were released using 100 mU PNGase F (EC 3.5.1.52; Roche Applied Science, Indianapolis, IN). N-Glycans were subsequently purified using C18 cartridges and graphitized carbon columns (both purchased from Alltech, Deerfield, IL). Permethylation was carried out according to Wedepohl et al. [37]. After the reaction, chloroform was added and the organic phase was washed with water until the pH of the water phase became neutral. The chloroform phase was finally removed under reduced atmosphere and the sample was dissolved in 75%-aqueous acetonitrile for MALDI-TOF-MS measurements.

### 2.4. Mass Spectrometry

MALDI-TOF mass spectra were recorded on an Ultraflex III mass spectrometer (Bruker Daltonics, Bremen, Germany) equipped with a Smartbeam laser and a LIFT-MS/MS facility. Each spectrum consisted of at least 2000 laser shots. Calibration was performed using a glucose ladder. Spectra were recorded in reflector positive ionization mode in the mass range of 1000–5000 Da. 0.5 *µ*L permethylated N-glycans was mixed on the ground steel target in a ratio of 1 : 1 with the matrix consisting of super DHB (10 mg/mL) dissolved in 10% aqueous acetonitrile. Baseline correction and peak picking were performed using Flexanalysis (Bruker Daltonics, Bremen, Germany). N-Glycan structures were annotated using GlycoPeakfinder and assigned N-glycan structures were generated with the GlycoWorkbench software.

### 2.5. Measurement of Serum CA125 and GLYCOV

CA125 was measured on a COBAS 6000 analyzer (Roche Diagnostics, Mannheim, Germany) using the CA125 II immunoassay. The cut-off value of CA125 was defined as 35 kU/L for pre- and postmenopausal women.

To calculate the GLYCOV score, relative areas of each N-glycan peak were rescaled to a total of 100%. The GLYCOV score was calculated for each sample according to Biskup et al. [28]. In short, the relative areas of the 11 N-glycan masses from the MALDI-TOF mass spectra that were revealed by the statistical analysis were combined into a quotient of the 7 upregulated glycan masses divided by the 4 downregulated glycan masses (sum of relative areas of *m*/*z* 3776.8, 3950.9, 4226.1, 4400.2, 4587.4, 4761.5, and 4935.7)/7∗4/(sum of relative areas of *m*/*z* 1579.7, 1783.8, 1987.9, and 2192.0). As serum samples were prepared and measured in duplicate, a middle GLYCOV value was built for each sample. Analyses were performed blindly without the knowledge of the results from CA125 measurements. GLYCOV and CA125 values are summarized in Table 2. The cut-off point for GLYCOV was earlier defined for the late-stage ovarian cancer samples as 0.63 (Mean + 2SD by 95% CI) [28].

### 2.6. Statistical Analysis

Data analyses were performed using SPSS version 21.0 (SPSS Inc, Chicago, IL). Mean, median, standard deviation and range were calculated for each group of patients with regard to FIGO and grading. The diagnostic accuracy of CA125 and GLYCOV marker was assessed by building of the receiver operating characteristic (ROC) curve at 95% confidence interval (CI) and calculation of the area under the curve (AUC). Binary logistic regression was carried out to evaluate the prediction accuracy of the marker to diagnose correctly the cohort of patients. Box plots were generated for the evaluation of the significance between the subcategories of patients. All box plots were performed after logarithmic transformation of the GLYCOV and CA125 values, which reduces skewness in the distribution of the results. *P* values lower than 0.05 were considered to be statistically significant.

## 3. Results

**Figure 1:**
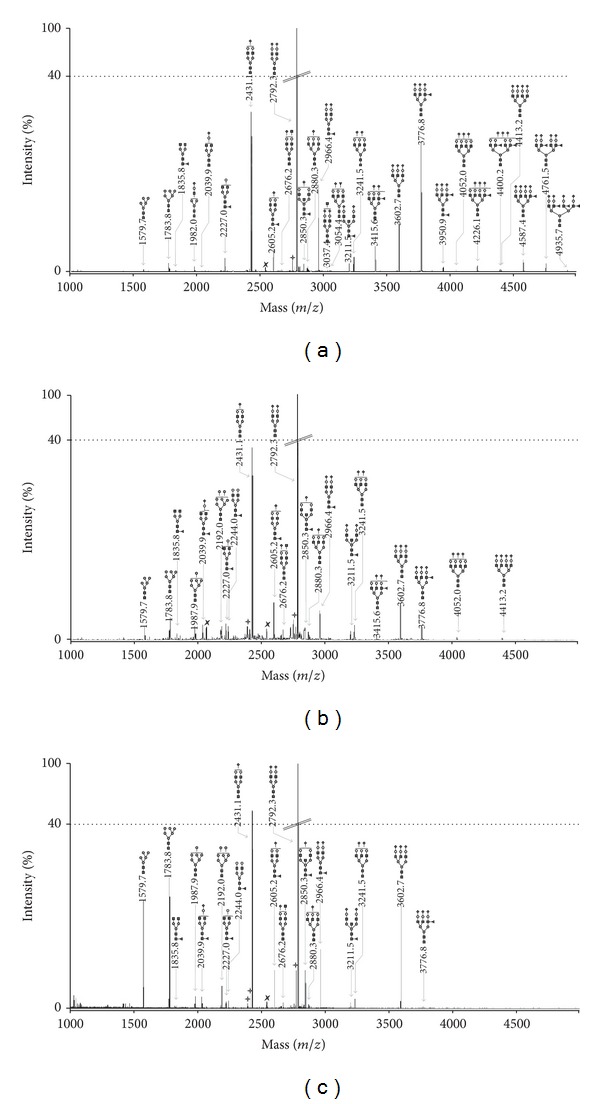
MALDI-TOF-MS spectra of permethylated N-glycans from (a) a patient with early-stage primary EOC, (b) a patient with benign ovarian disease, and (c) a healthy volunteer. Measurements were performed in the positive-ion mode. All ions are present in their sodiated form [M+Na]^+^. The monosaccharides are depicted as follows: Man, dark gray circle; Gal, light gray circle; GlcNAc, black square; Fuc, dark gray triangle; Neu5Ac, dark gray diamond; dark gray star polygon, underpermethylated; light gray star polygon, nonidentified.

**Figure 2:**
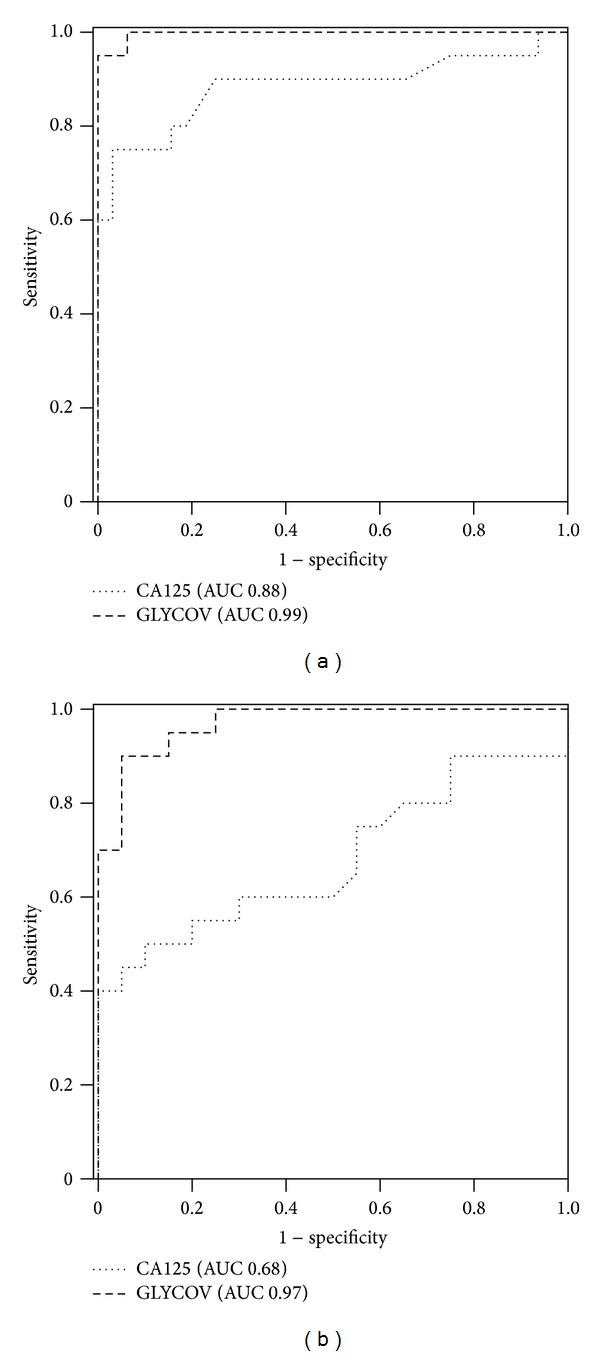
ROC curves for the GLYCOV and CA125 markers generated using 20 primary serous early-stage EOC patients and (a) 33 healthy controls or (b) 20 patients suffering from benign ovarian diseases.

The serum N-glycome was released using PNGase F from glycoproteins that had been denatured and carbamidomethylated prior to the enzymatic digestion. N-Glycans were isolated from serum proteins using C18 columns and were then desalted on carbograph cartridges. They were permethylated and finally measured with MALDI-TOF mass spectrometry. We were able to detect 47 N-glycan structures in the mass range of *m*/*z* 1000–5000 Da (Figure 1, Supplementary Data Table 1 available online at http://dx.doi.org/10.1155/2014/238197). The N-glycan structures of masses higher than *m*/*z* 3000 that correspond to sialylated fucosylated tri- and tetraantennary glycans are clearly upregulated in early-stage EOC patients (Figure 1(a)) when compared to patients suffering from benign ovarian diseases (Figure 1(b)) and healthy controls (Figure 1(c)). The downregulation of high-mannose structures Man_5–8_GlcNAc_2_ is visible at *m*/*z* 1579.8, 1783.9, 1988.0, and 2192.1 in the N-glycome of early-stage EOC patients when compared to patients suffering from benign ovarian diseases and healthy controls (Figure 1). The GLYCOV score was calculated for all the 73 patients included in this study from the relative areas obtained by MALDI-TOF-MS of the 11 N-glycan biomarkers as the quotient of upregulated to downregulated masses. Median GLYCOV value was 0.127 (range of 0.00–0.83) for the healthy control group, 0.380 (range of 0.02–1.27) for BOD patients, and 2.905 (range of 0.56–12.82) for preoperative early-stage EOC patients (Table 2). ROC curves were built for CA125 and GLYCOV markers for early-stage patients versus healthy controls (Figure 2(a)) and early-stage patients versus benign ovarian diseases (Figure 2(b)). The GLYCOV marker had a higher AUC value (0.992) than CA125 (0.884) to discriminate EOC patients from healthy controls. Interestingly, the discrimination between EOC patients and BOD patients was excellent with GLYCOV (AUC 0.970) whereas CA125 could only show low accuracy (AUC 0.680).

A binary logistic regression was then used to assess the specificity and the sensitivity of the GLYCOV and CA125 markers for each cohort (Table 3). GLYCOV values higher than the cut-off point of 0.63 were detected in 19 of 20 EOC patients (95% sensitivity) and only one of the 33 healthy controls was positive for GLYCOV (97% specificity) (Table 3(a)). One of the 33 healthy controls was positive for CA125 (97% specificity) and CA125 failed to detect 8/20 samples of primary EOC patients (60% sensitivity).

**Figure 3:**
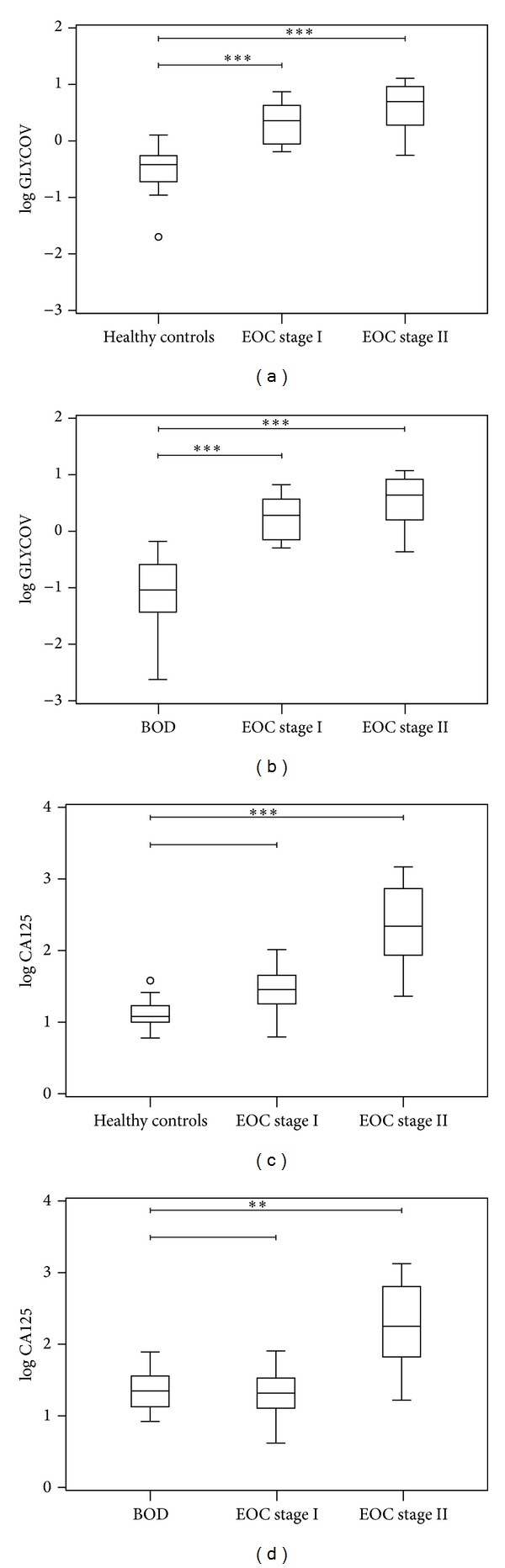
Box plots displays the comparison between GLYCOV (a, b) and CA125 (c, d) marker for early-stage serous EOC patients (FIGO stages I and II), patients with benign ovarian diseases and healthy controls. All values were transformed logarithmically to reduce the skewness in the distribution of the values. *P* values were calculated using the non-parametric Kruskal-Wallis pairwise comparison. *P* values lower than 0.05 are determined as statistically significant; **P < 0.01, and ***P < 0.001 are statistically significant at the given value.

**Figure 4:**
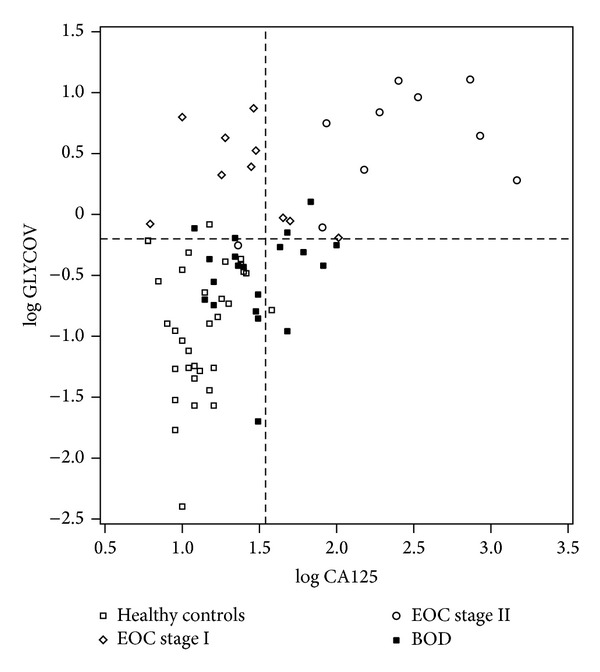
Two-dimensional scatter plot classifying the cohort enrolled in this study. The horizontal line of the plot represents the cut-off limit for GLYCOV [log⁡0.63 = −0.20] and the vertical line displays the limit for CA125 [log⁡35 = 1.54]. The scatter plot shows that the combination of CA125 and GLYCOV improves the diagnosis of early-stage primary EOC compared to when CA125 is used alone.

For the comparison between EOC and BOD patients, 16 BOD patients were correctly assigned with GLYCOV (80% specificity) whereas only 13 BOD patients were correctly predicted with CA125 (65% specificity) (Table 3(b)). When both markers are used in combination, the sensitivity was as good as the one of GLYCOV alone (95%) and the specificity reached 95%: only two of BOD patients (90% sensitivity) were still not recognized as such by both markers. Box plots were generated to demonstrate the ability of both markers to differentiate between early-stage EOC, BOD patients and healthy controls. Interestingly, GLYCOV could discriminate significantly between stage I EOC patients, stage II EOC patients and BOD patients as well as between stage I EOC patients, stage II EOC patients and healthy controls (Figures 3(a) and 3(b)). On the contrary, CA125 could not distinguish between stage I EOC patients, BOD patients, and healthy controls but it was able to detect stage II EOC patients from healthy controls (*P* < 0.001) and from BOD patients (*P* < 0.05) (Figures 3(c) and 3(d)). The separation between the grading stages was not statistically significant for both CA125 and GLYCOV markers (data not shown). Finally, data were visualized with a two-dimensional scatter plot (Figure 4), in which the threshold for GLYCOV was placed at the cut-off point 0.63 and the threshold for CA125 was placed at the cut-off point 35 kU/L. The GLYCOV values showed no significant differences between both stages and only one of the 20 early-stage EOC samples had a value below the threshold. The scatter plot shows that the GLYCOV marker supports the CA125 marker in diagnosis of primary EOC patients at an early-stage of the disease and provides a better prediction for BOD patients.

## 4. Discussion

In this work, we measured the permethylated N-glycome derived from human serum using MALDI-TOF mass spectrometry to investigate whether or not GLYCOV could detect early-stage EOC patients and differentiate malignant from benign tumors. The downregulation of high-mannose structures and the upregulation of sialylated tri- and tetraantennary structures containing one or more fucoses could be observed in samples both in stage I EOC patients (9/10) and in stage II EOC patients (10/10). It should be noted that these glycan features were absent in most of the BOD samples (16/20).

The downregulation of high-mannose structures in ovarian cancer patients reported here is corroborated by reports of other groups who used different analytical workflows and mass spectrometry techniques [38–40]. Decrease in high-mannose N-glycans was recently observed also in gastric cancer patients but this form of cancer was accompanied by a decrease in complex-type monoantennary N-glycans and an increase of agalactosylated biantennary N-glycans, which was not observed here [31]. On the contrary, elevation of high-mannose N-glycans was measured in the serum of breast cancer patients by the same group [41]. The modulation of high-mannose content probably stems from complement C3, the only acute-phase protein, which carries high-mannose N-glycans [42]. The increase of the monofucosylated triantennary trisialylated N-glycan at *m*/*z* 3776.9, carrying a sialyl Lewis^X^ antigen, that was reported here is a common feature in several cancers and inflammatory conditions: prostate metastatic cancer [43], lung cancer [44], breast cancer [27], acute pancreatitis, and sepsis [29]. The sialyl Lewis^X^ antigen is carried by acute-phase glycoproteins that circulate in serum of patients suffering from inflammatory conditions: *α*-1 acid glycoprotein, haptoglobin, and *α*1-antichymotrypsin [45]. In addition, the upregulation of the corresponding glycosyltransferases was also measured in the serum of ovarian cancer patients: (*α*1-3) fucosyltransferase [46], (*β*1-4) galactosyltransferase [47], and (*α*2-6) sialyltransferase [48, 49].

The mechanisms explaining aberrant glycan modulations observed in the sera of EOC patients correlate with modulations of the glycosylation machinery occurring in the liver as acute-phase glycoproteins that are synthesized by hepatocytes. The production of acute-phase proteins is generated by different stimuli involving cytokines (TNF, IL-1, and IL-6), which are secreted by ovarian tumor cells* in vitro* and* in vivo* [50, 51]. Using hepatoma cell lines, Van Dijk and Mackiewicz showed that cytokines induce glycosylation modulations of acute-phase glycoproteins, the observed changes being independent of their synthesis rate [52].

In this work, we showed that the GLYCOV marker allowed distinguishing early-stage EOC patients from healthy volunteers with a sensitivity (95%) that was 35% better than that of CA125. Remarkably, GLYCOV was particularly more efficient (95% sensitivity and 80% specificity) than CA125 (60% sensitivity and 65% specificity) to distinguish early-stage EOC from BOD patients, which is of high relevance for gynecologic oncologists because the diagnosis of malignancy in adnexal masses is difficult prior to surgery as CA125 is expressed in only about 50% of early-stage tumors [12, 53]. While both markers were statistically significant for the discrimination between the healthy control group and the EOC patients at stage II, only the GLYCOV value showed significant differences for the primary stage I EOC samples, where CA125 failed.

## 5. Conclusion

Our data suggests that the power of the glycan marker GLYCOV to discriminate early-stage EOC from healthy control and from benign diseases is significant better than that of CA125. As glycan modulations are observed in patient serum as early as in FIGO stage I, it appears that serum glycome modulations are initiated at the onset of EOC and therefore could be used as an early diagnostic marker that is independent from CA125, which is synthesized by tumor cells. As our study was limited to a restricted number of samples, future studies should incorporate a larger cohort of patients including other histological types and menopausal statuses.

## Supplementary Material

Supplementary Data Table 1: N-glycan structures identified by MALDI-TOF-MS Average areas were calculated for each glycan mass for healthy controls, benign ovarian disease and EOC-patients.

## Figures and Tables

**Table 1:** Primary serous epithelial ovarian cancer patient demographics.

	Healthy controls	BOD patients	EOC patients
Number of patients	33	20	20
Age			
Mean	52	57	57
Median	50	52	58
Range	40–81	35–97	40–78
SD	10.08	14.36	10.46
Stage			
I	—	—	10
II	—	—	10
Grade			
1	—	—	1
2	—	—	10
3	—	—	9
GLYCOV value∗			
Mean	0.21	0.42	4.31
Median	0.13	0.38	2.90
Range	0.00–0.83	0.02–1.27	0.56–12.82
SD	0.21	0.29	3.84
CA125 (kU/L)			
Mean	14.45	36.90	225.86
Median	12.00	30.50	65.50
Range	6–38	12–100	6–1474
SD	6.92	24.36	375.87

∗GLYCOV values were calculated using the relative areas obtained from the MALDI-TOF-MS spectra: (sum of relative areas of *m*/*z* 3776.8, 3950.9, 4226.1, 4400.2, 4587.4, 4761.5, 4935.7)/7∗4/ (sum of relative areas of *m*/*z* 1579.7, 1783.8, 1987.9 and 2192.0).

**Table 2:** Preoperative serum GLYCOV and CA125 values for primary serous epithelial ovarian cancer patients, patients suffering from benign diseases and healthy controls.

	GLYCOV value			CA125		
	Mean	Median	Range	Mean	Median	Range
Controls	0.21	0.13	0–0.83	14.45	12.0	6–38
BOD	0.42	0.38	0.02–1.27	36.90	30.50	12–100
EOC patients	4.31	2.90	0.56–12.82	225.86	65.50	6–1474
FIGO stage						
I	2.92	2.29	0.64–7.44	33.82	28.5	6–103
II	5.70	5.01	0.56–12.82	417.9	221.0	23–1474
Grade						
I + II	4.54	3.35	0.64–12.82	259.0	30.0	6–1474
III	4.03	2.33	0.56–12.52	185.11	86	18–851

**(a):** 

	Prediction model		
	EOC/healthy		Sensitivity/specificity
	0	1	
GLYCOV			
EOC	1	19	0.95
Healthy controls	32	1	0.97
CA125			
EOC	8	12	0.60
Healthy controls	32	1	0.97
Combined			
EOC	1	19	0.95
Healthy controls	32	1	0.97

**(b):** 

	Prediction model		
	EOC/BOD		Sensitivity/specificity
	0	1	
GLYCOV			
EOC	1	19	0.95
BOD	16	4	0.80
CA125			
EOC	8	12	0.60
BOD	13	7	0.65
Combined			
EOC	1	19	0.95
BOD	18	2	0.90

## Abbreviations

AUC: Area Under The Curve
BOD: Benign Ovarian Diseases
CA125: Cancer Antigen 125
CI: Confidence Interval
EOC: Epithelial Ovarian Cancer
FIGO: International Federation of Obstetricians and Gynaecologists
GLYCOV: Glycan-Based Biomarker Developed by The Authors to Diagnose Ovarian Cancer
MALDI-TOF: Matrix-Assisted Laser Desorption Ionization Time-of-Flight
PNGase F: Peptide-N4-(N-Acetyl-*β*-glucosaminyl) Asparagine Amidase F
ROC: Receiver Operating Characteristic.
