# Supplementary material for: The Serum Glycome to Discriminate between Early-Stage Epithelial Ovarian Cancer and Benign Ovarian Diseases
Source: Dis Markers. 2014 Aug 12;2014:238197. doi: 10.1155/2014/238197 (PMC4145549; doi:10.1155/2014/238197)
Supplement: Supplementary file 1 — Supplementary Data Table 1: N-glycan structures identified by MALDI-TOF-MS Average areas were calculated for each glycan mass for healthy controls, benign ovarian disease and EOC-patients. [file 238197.f1.doc]

| mass [permeth., Na^+^] | Formula | Structure | average area (healthy) | average area (benign) | average area (EOC) |  |
| --- | --- | --- | --- | --- | --- | --- |
| 1416.6 | HexNAc_3_Hex_3_ |  | 0.1642 | 0.1210 | 0.0593 | 1 |
| 1579.7 | HexNAc_2_Hex_5_ |  | 1.1851 | 0.6700 | 0.3314 | 2 |
| 1620.7 | HexNAc_3_Hex_4_ |  | 0.1522 | 0.1850 | 0.0542 | 3 |
| 1661.8 | HexNAc_4_Hex_3_ |  | 0.0554 | 0.1180 | 0.0318 | 4 |
| 1783.8 | HexNAc_2_Hex_6_ |  | 1.7007 | 1.4470 | 0.5974 | 5 |
| 1835.8 | HexNAc_4_Hex_3_dHex_1_ |  | 0.8064 | 0.4870 | 0.3086 | 6 |
| 1981.9 | HexNAc_3_Hex_4_Neu5Ac_1_ |  | 0.6715 | 0.6990 | 0.4450 | 7 |
| 1987.9 | HexNAc_2_Hex_7_ |  | 0.3106 | 0.5065 | 0.1220 | 8 |
| 2039.9 | HexNAc_4_Hex_4_dHex_1_ |  | 1.1598 | 0.9055 | 0.2788 | 9 |
| 2070.0 | HexNAc_4_Hex_5_ |  | 0.4194 | 0.6385 | 0.2913 | 10 |
| 2111.0 | HexNAc_5_Hex_4_ |  | 0.2119 | 0.4450 | 0.1755 | 11 |
| 2186.0 | HexNAc_3_Hex_5_Neu5Ac_1_ |  | 0.3424 | 0.6405 | 0.1719 | 12 |
| 2192.0 | HexNAc_2_Hex_8_ |  | 0.6548 | 1.2665 | 0.3543 | 13 |
| 2227.0 | HexNAc_4_Hex_4_Neu5Ac_1_ |  | 1.0522 | 1.4475 | 0.9234 | 14 |
| 2244.0 | HexNAc_4_Hex_5_dHex_1_ |  | 0.5801 | 0.7665 | 0.1741 | 15 |
| 2285.0 | HexNAc_5_Hex_4_dHex_1_ |  | 0.2016 | 0.4945 | 0.2355 | 16 |
| 2315.1 | HexNAc_5_Hex_5_ |  | 0.0427 | 0.2960 | 0.0734 | 17 |
| 2390.1 | HexNAc_3_Hex_6_Neu5Ac_1_ |  | 0.0493 | 0.5605 | 0.1038 | 18 |
| 2431.1 | HexNAc_4_Hex_5_Neu5Ac_1_ |  | 14.7271 | 16.9540 | 12.4294 | 19 |
| 2489.1 | HexNAc_5_Hex_5_dHex_1_ |  | 0.2489 | 0.6930 | 0.2055 | 20 |
| 2519.1 | HexNAc_5_Hex_6_ |  | 0.0125 | 0.2775 | 0.0623 | 21 |
| 2605.2 | HexNAc_4_Hex_5_Neu5Ac_1_dHex_1_ |  | 2.3822 | 3.4220 | 1.5189 | 22 |
| 2663.2 | HexNAc_5_Hex_5_dHex_2_ |  | 0.2341 | 0.5660 | 0.1577 | 23 |
| 2676.2 | HexNAc_5_Hex_5_Neu5Ac_1_ |  | 0.7468 | 1.3330 | 0.5757 | 24 |
| 2693.2 | HexNAc_5_Hex_6_dHex_1_ |  | 0.0125 | 0.2500 | 0.0557 | 25 |
| 2764.3 | HexNAc_6_Hex_6_ |  | 0.7471 | 0.7070 | 0.5350 | 26 |
| 2792.3 | HexNAc_4_Hex_5_Neu5Ac_2_ |  | 57.5563 | 48.6340 | 59.3607 | 27 |
| 2850.3 | HexNAc_5_Hex_5_Neu5Ac_1_dHex_1_ |  | 1.5135 | 1.3510 | 1.1118 | 28 |
| 2880.3 | HexNAc_5_Hex_6_Neu5Ac_1_ |  | 0.5421 | 0.5065 | 0.4684 | 29 |
| 2966.4 | HexNAc_4_Hex_5_Neu5Ac_2_dHex_1_ |  | 3.5901 | 2.9680 | 3.7079 | 30 |
| 3037.4 | HexNAc_5_Hex_5_Neu5Ac_2_ |  | 0.1162 | 0.2375 | 0.1183 | 31 |
| 3054.4 | HexNAc_5_Hex_6_Neu5Ac_1_dHex_1_ |  | 0.1066 | 0.2720 | 0.1480 | 32 |
| 3211.5 | HexNAc_5_Hex_5_Neu5Ac_2_dHex_1_ |  | 0.9229 | 0.9490 | 1.0335 | 33 |
| 3241.5 | HexNAc_5_Hex_6_Neu5Ac_2_ |  | 1.1705 | 1.1450 | 1.4503 | 34 |
| 3415.6 | HexNAc_5_Hex_6_Neu5Ac_2_dHex_1_ |  | 0.2721 | 0.5075 | 0.5740 | 35 |
| 3602.7 | HexNAc_5_Hex_6_Neu5Ac_3_ |  | 3.7263 | 4.2375 | 6.3063 | 36 |
| 3690.7 | HexNAc_6_Hex_7_Neu5Ac_2_ |  | 0.1096 | 0.2750 | 0.1292 | 37 |
| 3776.8 | HexNAc_5_Hex_6_Neu5Ac_3_dHex_1_ |  | 0.9319 | 1.9120 | 4.3444 | 38 |
| 3864.8 | HexNAc_6_Hex_7_Neu5Ac_2_dHex_1_ |  | 0.0087 | 0.0965 | 0.0717 | 39 |
| 3950.9 | HexNAc_5_Hex_6_Neu5Ac_3_dHex_2_ |  | 0.0128 | 0.1375 | 0.0607 | 40 |
| 4052.0 | HexNAc_6_Hex_7_Neu5Ac_3_ |  | 0.1190 | 0.2640 | 0.2089 | 41 |
| 4226.1 | HexNAc_6_Hex_7_Neu5Ac_3_dHex_1_ |  | 0.0282 | 0.1480 | 0.0938 | 42 |
| 4400.2 | HexNAc_6_Hex_7_Neu5Ac_3_dHex_2_ | 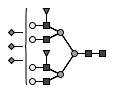 | 0.0130 | 0.0390 | 0.0334 | 43 |
| 4413.2 | HexNAc_6_Hex_7_Neu5Ac_4_ | 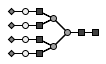 | 0.0859 | 0.2635 | 0.2722 | 44 |
| 4587.4 | HexNAc_6_Hex_7_Neu5Ac_4_dHex_1_ | 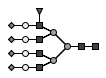 | 0.0265 | 0.1355 | 0.1775 | 45 |
| 4761.5 | HexNAc_6_Hex_7_Neu5Ac_4_dHex_2_ | 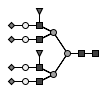 | 0.0028 | 0.0165 | 0.0496 | 46 |
| 4935.7 | HexNAc_6_Hex_7_Neu5Ac_4_dHex_3_ | 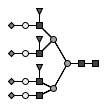 | 0.0000 | 0.0030 | 0.0035 | 47 |

**Supplementary Data Table 1.** N-glycan structures identified by MALDI-TOF-MS Average areas were calculated for each glycan mass for healthy controls, benign ovarian disease and EOC-patients.
